# Metabolic Profiling of Bladder Cancer Patients’ Serum Reveals Their Sensitivity to Neoadjuvant Chemotherapy

**DOI:** 10.3390/metabo12060558

**Published:** 2022-06-17

**Authors:** Juntao Zhuang, Xiao Yang, Qi Zheng, Kai Li, Lingkai Cai, Hao Yu, Jiancheng Lv, Kexin Bai, Qiang Cao, Pengchao Li, Haiwei Yang, Junsong Wang, Qiang Lu

**Affiliations:** 1 Department of Urology, The First Affiliated Hospital of Nanjing Medical University, Nanjing 210029, China; zhuangjt2020@foxmail.com (J.Z.); yangxiao2915@163.com (X.Y.); kaili98@njmu.edu.cn (K.L.); lingkaicai1996@163.com (L.C.); yh2838@163.com (H.Y.); doctorjiancheng@sina.com (J.L.); 13210612019@163.com (K.B.); qiang_cao@126.com (Q.C.); superkulian@aliyun.com (P.L.); haiweiyang@njmu.edu.cn (H.Y.); 2 Center of Molecular Metabolism, Nanjing University of Science and Technology, Nanjing 210094, China; zhengqi@njust.edu.cn

**Keywords:** metabolomics, serum, neoadjuvant chemotherapy, bladder cancer, ^1^H-NMR, UPLC-MS

## Abstract

Numerous patients with muscle-invasive bladder cancer develop low responsiveness to cisplatin. Our purpose was to explore differential metabolites derived from serum in bladder cancer patients treated with neoadjuvant chemotherapy (NAC). Data of patients diagnosed with cT2-4aNxM0 was collected. Blood samples were retained prospectively before the first chemotherapy for untargeted metabolomics by ^1^H-NMR and UPLC-MS. To identify characterized metabolites, multivariate statistical analyses were applied, and the intersection of the differential metabolites discovered by the two approaches was used to identify viable biomarkers. A total of 18 patients (6 NAC-sensitive patients and 12 NAC-resistant patients) were enrolled. There were 29 metabolites detected by ^1^H-NMR and 147 metabolites identified by UPLC-MS. Multivariate statistics demonstrated that in the sensitive group, glutamine and taurine were considerably increased compared to their levels in the resistant group, while glutamate and hypoxanthine were remarkably decreased. Pathway analysis and enrichment analysis showed significant alterations in amino acid pathways, suggesting that response to chemotherapy may be related to amino acid metabolism. In addition, hallmark analysis showed that DNA repair played a regulatory role. Overall, serum metabolic profiles of NAC sensitivity are significantly different in bladder cancer patients. Glycine, hypoxanthine, taurine and glutamine may be the potential biomarkers for clinical treatment. Amino acid metabolism has potential value in enhancing drug efficacy.

## 1. Introduction

Bladder cancer is one of the most prevalent malignant tumours worldwide, with about 550,000 new cases reported annually [1]. When the malignant tumour breaks through the lamina propria and invades the muscle tissues, it is considered as muscle-invasive bladder cancer (MIBC). MIBC is prone to recurrence and metastasis, and has a poor prognosis, with 5-year overall survival (OS) of 36–48% [2]. At present, the standard treatment for patients with MIBC is neoadjuvant chemotherapy (NAC) followed by radical cystectomy (RC) and pelvic lymph node dissection [3]. Several studies have shown that patients with T2-T4aN0M0 bladder cancer treated with NAC had a better oncology outcome compared to RC alone. Specifically, addition of NAC increased 5-year OS by 8% relative to its absence [4], and cisplatin-based NAC resulted in better OS and cancer specific survival (CSS) outcomes compared to other chemotherapy drugs [5].

Unfortunately, not all patients respond to NAC, with a modest pathological complete response (pCR) of 25.7–38% [4,6], and many patients still suffer from delays in treatment due to chemotherapy intolerance. Therefore, learning how to predict the efficacy of NAC is crucial to guide clinical strategy. Pathological response is an objective criterion, while invasive surgery is required to obtain tissues. Molecular subtypes have demonstrated a contradictory result in the assessment of NAC sensitivity and OS, especially in basal and luminal subtypes [7,8]. In addition, bladder cancer patients with ERCC2 mutations are more sensitive to cisplatin-based NAC [9,10]. Liquid biopsy has been widely explored in recent years because of its convenience and low invasiveness. Circulating tumour DNA (ctDNA) and circulating tumour cells (CTCs) have great potential in predicting the efficacy of NAC in MIBC patients [11,12], and our previous study also confirmed the value of CTCs in predicting NAC sensitivity [13].

Serum metabolomics is essentially part of liquid biopsy. At present, serum metabolomics has achieved excellent results in the exploration of risk factors for tumour recurrence and biomarkers for tumour diagnosis, such as endometrial cancer, breast cancer and prostate cancer [14,15,16]. In addition, the correlations between differential metabolites and NAC for advanced patients are also emerging. Metabolic profile alterations before and after NAC, as well as predictive models of NAC efficacy based on metabolites, have been widely reported for colorectal cancer, oesophageal cancer, breast cancer and lung cancer [17,18,19,20,21].

With regard to bladder cancer, current studies have concentrated on metabolites that can predict bladder tumour recurrence or differentiate bladder cancer patients from non-tumour populations [22]. However, metabolomics for predicting drug efficacy of bladder cancer is rarely reported. In the present study, we focused on identifying metabolic biomarkers for predicting sensitivity of NAC by using nuclear magnetic resonance (^1^H-NMR) and ultra-performance liquid chromatography-mass spectrometry (UPLC-MS), then integrated them to explore potential metabolic pathways and molecular targets using bioinformatics.

## 2. Results

### 2.1. Characteristics of the Patients

A total of 18 MIBC patients were selected, with six patients identified as NAC-sensitive and 12 patients assessed as NAC-resistant. The details are shown in Table 1. There were no significant differences in gender, age, BMI, smoking history and chemical exposure history between the two groups.

### 2.2. Serum Metabolome Spectrum

#### 2.2.1. Metabolome Spectrum for ^1^H-NMR

**Figure 1:**
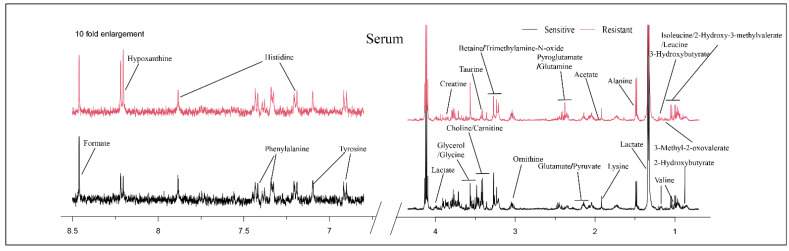
Typical 500 MHz ^1^H-NMR spectra of serum for the two groups. Red spectra represent NAC-resistant group and black spectra represent NAC-sensitive group.

Typical NMR spectra of metabolites between the sensitive and resistant groups are shown in Figure 1. A total of 29 metabolites were identified in the serum, including 2-hydroxybutyrate, isoleucine, 2-hydroxy-3-methylvalerate, leucine, valine, 3-methyl-2-oxovalerate, 3-hydroxybutyrate, lactate, alanine, lysine, acetate, glutamate, pyruvate, pyroglutamate, glutamine, ornithine, choline, carnitine, betaine, trimethylamine-N-oxide, taurine, glycerol, glycine, creatine, tyrosine, histidine, phenylalanine, hypoxanthine and formate.

#### 2.2.2. Metabolome Spectrum for UPLC-MS

In order to overcome the issue of the small number of compounds detected by ^1^H-NMR [23], the same samples were evaluated by UPLC-MS, a technology with the advantages of high sensitivity and broad metabolite obtainment. A total of 262 compounds were detected, and 147 of them were identified as human metabolites according to The Human Metabolome Database (HMDB) [24].

### 2.3. Multivariate Statistical Analysis

#### 2.3.1. PCA and OPLS-DA Analysis for ^1^H-NMR

**Figure 2:**
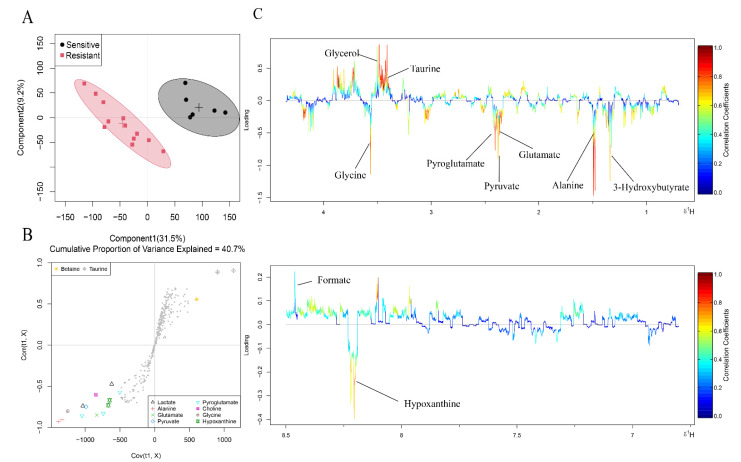
OPLS-DA analysis of the data obtained from ^1^H-NMR between the two groups. (**A**) Score plots. Each point is one sample. Different groups are in different colours, with circles representing the 95% confidence interval. (**B**) S plots. Different shapes represent different metabolites. (**C**) Corresponding colour-coded loading plots. Colour is encoded by the absolute correlation coefficient of each variable to the grouping, with hot-colour more significant than cool-colour signals.

Metabolomics usually uses multivariate statistics to further explore metabolite differences between two groups. The principal component analysis (PCA) score plot showed partial overlap between the two groups (Figure S1). In order to better identify the differences, we used the orthogonal partial least squares discriminant analysis (OPLS-DA) method to perform a comprehensive evaluation. In the OPLS-DA score plot (Figure 2A), the sensitive group was far away from the resistant group, indicating metabolic disturbances between the two groups. S-plots (Figure 2B) and color-coded loading plots (Figure 2C) were used to visualize the contribution of variables between the two groups.

Fold change and *p* value are shown in Table 2. In the sensitive group, glutamine and taurine were considerably increased compared to their levels in the resistant group, while 2-hydroxy-3-methylvalerate, 3-methyl-2-oxovalerate, 3-hydroxybutyrate, alanine, glutamate, pyruvate, pyroglutamate, glycine and hypoxanthine were remarkably decreased. Therefore, metabolite levels in patients with or without NAC response were different.

#### 2.3.2. PCA and OPLS-DA Analysis for UPLC-MS

As previously stated, PCA and OPLS-DA were used for multivariate analysis of the metabolites obtained from UPLC-MS. Consistent with ^1^H-NMR data, the PCA score plot (Figure S2A) showed partially overlapping, while the OPLS-DA (Figure S2B) score plot showed well distinguished differences between the two groups.

**Figure 3:**
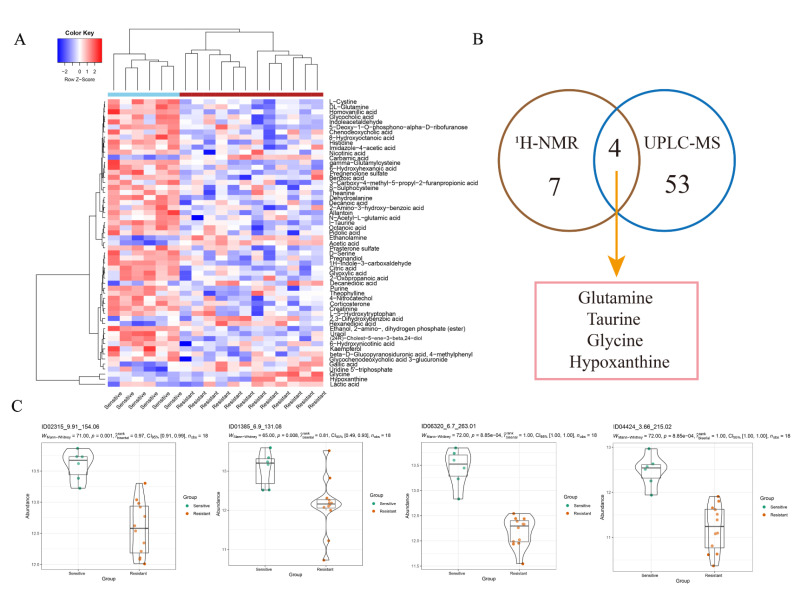
OPLS-DA analysis of the data obtained from UPLC-MS between the two groups. (**A**) Differential metabolites identified by UPLC-MS. Colours from blue to red indicate the relative intensity of the metabolites in the two groups. (**B**) Venn diagram shows the metabolites detected by both methods. (**C**) Other significant metabolites identified by UPLC-MS between the two groups.

A total of 57 metabolites were considered as differential metabolites (Figure 3A). Glutamine, taurine, glycine and hypoxanthine were detected in both methods with the same trends (Figure 3B). These four metabolites may be more reliable biomarkers for predicting the NAC response in bladder cancer. In addition, some significant metabolites that were not obtained by ^1^H-NMR were identified by UPLC-MS due to its high sensitivity, including glyoxylic acid, ornithine, L-cystine, purine, uracil, serine and histidine, etc. (Figure 3C and  Figure S3). Metabolite names corresponding to each violin diagram are shown in Table S2.

### 2.4. Metabolic Pathway Analysis

**Figure 4:**
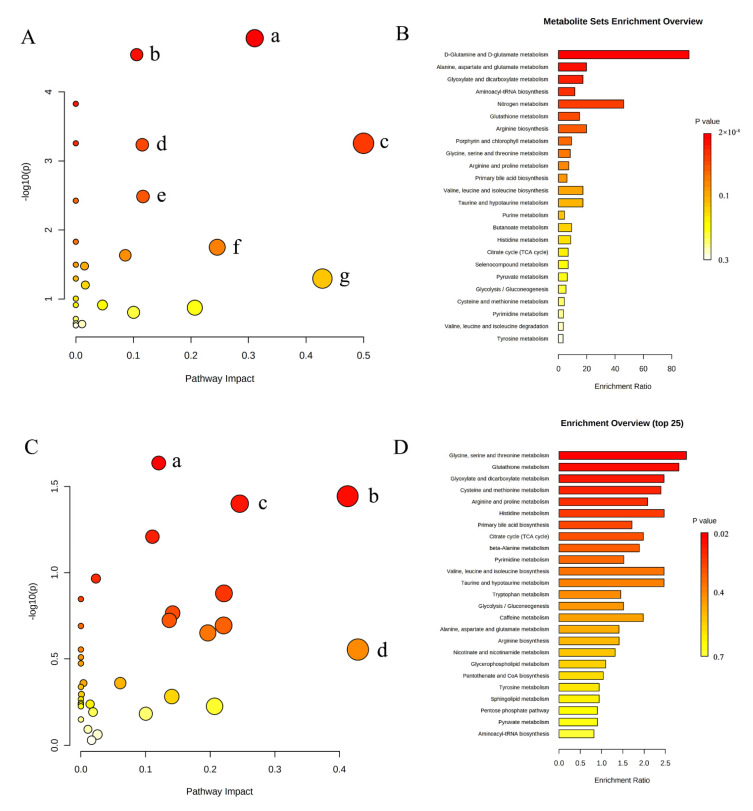
Summary of pathway analysis and MSEA. (**A**) Pathway analysis of metabolites identified by ^1^H-NMR. (a) Alanine, aspartate and glutamate metabolism; (b) glyoxylate and dicarboxylate metabolism; (c) D-glutamine and D-glutamate metabolism; (d) glutathione metabolism; (e) arginine biosynthesis; (f) glycine, serine and threonine metabolism; (g) taurine and hypotaurine metabolism. (**B**) MSEA of metabolites identified by ^1^H-NMR. (**C**) Pathway analysis of metabolites identified by UPLC-MS. (a) Glutathione metabolism; (b) glyoxylate and dicarboxylate metabolism; (c) glycine, serine and threonine metabolism; (d) taurine and hypotaurine metabolism. (**D**) MSEA of metabolites identified by UPLC-MS.

We performed pathway analysis using Metaboanalyst5.0 (https://www.metaboanalyst.ca/ (accessed on 16 February 2022)) to explore potential metabolic pathways that might affect NAC sensitivity. Differential metabolites with *p* < 0.05 were selected to import into the tool. Figure 4A indicates the pathway analysis results of metabolites detected by ^1^H-NMR, and the detailed results are shown in Table 3. MSEA shows that amino acid metabolism pathway and carbohydrate metabolism pathway were significantly enriched, including alanine, aspartate and glutamate metabolism, glyoxylate and dicarboxylate metabolism, D-glutamine and D-glutamate metabolism, glutathione metabolism, arginine biosynthesis, glycine, serine and threonine metabolism (Figure 4B).

The results of pathway analysis (Figure 4C and Table 4) and MSEA (Figure 4D) of characteristic metabolites identified by UPLC-MS were similar to the results of ^1^H-NMR. Glutathione metabolism, glyoxylic acid and dicarboxylic acid metabolism, glycine, serine and threonine metabolism remained remarkably enriched. These results suggest that the three metabolic pathways may be the target pathways related to chemotherapy sensitivity.

### 2.5. Potential Proteins and Genes Associated with Metabolites

**Figure 5:**
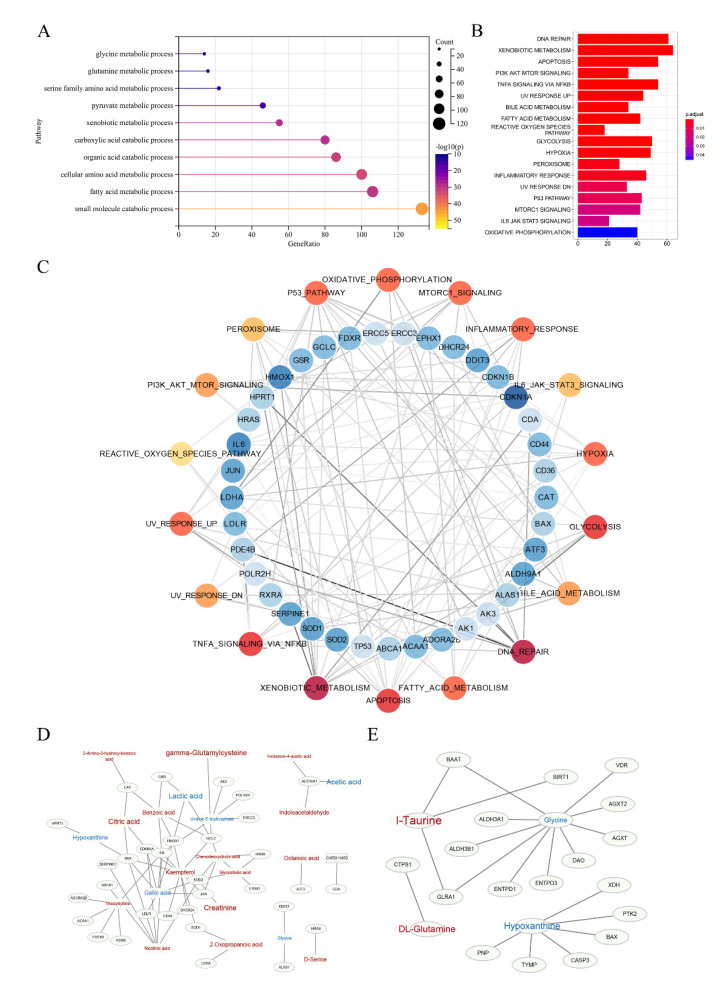
Enrichment analysis based on the self-built database, which integrated metabolites and proteins. (**A**) Summary of GO enrichment analysis. (**B**) Hallmark analysis of metabolites identified by UPLC-MS. (**C**) Detailed network diagram of genes related to pathways. Red dots represent pathways and blue dots represent genes. The darker the colour, the greater the degree. The shade of the line colour indicates edge betweenness. (**D**,**E**) The network of these significant genes and their related metabolites, and the four potential biomarkers and their related genes. Red represents the metabolites increasing in the sensitive group compared to the resistant group, while blue represents decreasing. The font size represents its significance.

In order to search for the potential molecular target, we further performed bioinformatics analysis through a self-built database that integrated metabolites and proteins. The interacting proteins were traced according to the characteristic metabolites, and their corresponding genes were enriched. Due to the limited results of ^1^H-NMR detection, we only selected the abundant metabolites detected in UPLC-MS for the analysis of gene and protein level. First, GO enrichment analysis demonstrated that there were significant alterations in amino acid pathways and organic acid transport (Figure 5A). Then, we use hallmark analysis to show the signalling pathways. There were 18 remarkable pathways (Figure 5B) enriched by the metabolites obtained from UPLC-MS, indicating that alterations in these pathways may affect the sensitivity of NAC. The genes associated with these pathways are detailed in Figure S4. Then, we used cystoscope to analyse the network, finding 37 genes with strong potential correlation on the basis of degree of node table over 3 (Figure 5C), including BAX, CDKN1A, SOD1 and ERCC3. Metabolites directly associated with these genes are shown in Figure 5D. Finally, we synthesized the results of GO analysis and Hallmark analysis, and obtained the network diagram of the relationship between these four significant metabolites and their related genes (Figure 5E).

## 3. Discussion

**Figure 6:**
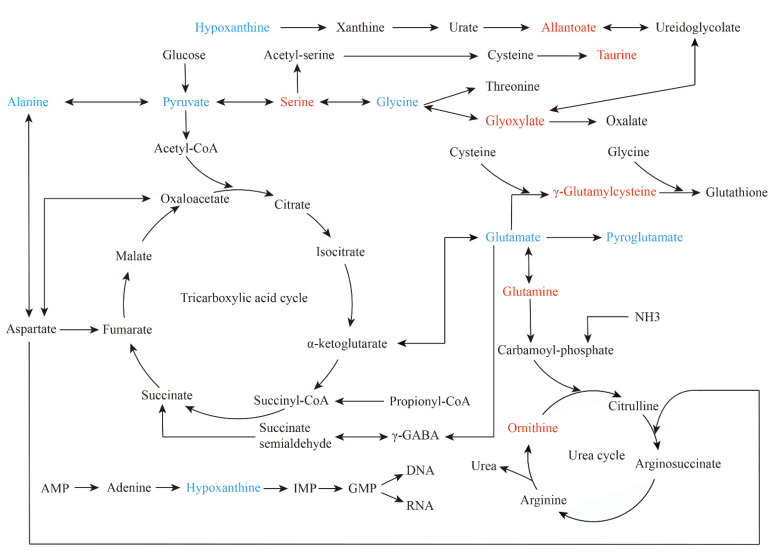
Summary of significant metabolic pathways about sensitivity to NAC in bladder cancer. It is mapped according to KEGG pathway (https://www.genome.jp/kegg/pathway.html (accessed on 11 April 2022)). Red represents elevated metabolites in the sensitive group, and blue represents reduced metabolites.

Metabolites represent the lowest level of biological information, integrating the changes of gene, transcription and protein [25]. Metabolomics establishes new opportunities for identifying cancer risk factors, distinguishing biomarkers for cancer monitoring and discovering drugs targeted to cancer metabolism [23]. In this pilot study, we survey a metabolic landscape associated with NAC sensitivity in patients with MIBC by ^1^H-NMR and UPLC-MS (Figure 6). Some characteristic metabolites about amino acids, organic acids, purines and ketone bodies were obtained. Then, we used bioinformatics methods to visualize the internal metabolic pathways and potential molecular pathways under metabolic phenotypes.

^1^H-NMR, a mature metabolomics technique, is known for its unbiasedness, robustness and complete database [26]. Liquid chromatography has the advantage of high sensitivity and great resolution, applying to the detection of heat-resistant and non-volatile compounds [27]. However, no single metabolomics platform could present a complete metabolic profile. Therefore, we used complementary approaches to enhance the coverage of metabolites to demonstrate the metabolic profiles of bladder cancer patients treated with NAC and obtained biomarkers with higher potential by taking the intersection of the two outcomes.

With respect to our results, glycine and hypoxanthine were significantly decreased, while taurine and glutamine were significantly increased in sensitive patients. Among breast cancer studies, glycine decreased significantly after chemotherapy in chemotherapy-sensitive patients, but not in resistant patients [28]. That is consistent with the results of neoadjuvant chemoradiotherapy for oesophageal cancer, in which the levels of glycine and serine were lower in sensitive patients [18]. Several studies have shown that dietary therapy limiting the intake of serine and glycine can play a certain anti-tumour role [29,30]. The decrease in intracellular nucleotide concentration could improve the efficacy of gemcitabine [31], while glycine is a precursor to de novo synthesis of purine nucleotides [32]. Therefore, there may be a synergistic effect between lower glycine levels and efficacy of cisplatin or gemcitabine. In bladder cancer, increased glycine has been found, compared with patient health [33]. Taurine is a valuable metabolite in urine of bladder cancer patients, and is elevated in ill patients [34]. Metabolic disorders of taurine are also associated with recurrence of NMIBC [35]. Additionally, our study found that taurine is also a potential biomarker in serum. Taurine combined with cisplatin could enhance the inhibitory effect of cisplatin on the proliferation of cervical cancer cells by up-regulating P53 expression and down-regulating anti-apoptotic protein expression [36,37]. Therefore, a high level of taurine may contribute to the effect of chemotherapy. Hypoxanthine, an upstream metabolite in the nucleotide biosynthetic pathway, was considerably decreased in sensitive patients, probably related to DNA damage repair. In NAC-sensitive breast cancer patients, hypoxanthine decreased significantly after chemotherapy [21]. In low-risk bladder cancer patients, elevated hypoxanthine in urine predicts the likelihood of recurrence [38]. In addition, lipids are also important metabolites. Yang, et al. [17] investigated the plasma of colorectal cancer patients and found that nine metabolites, primarily lipids, could predict the sensitivity to NAC. In our study, we also identified some lipids, such as PA (14:0/20:2(11Z,14Z)) and PE (14:0/22:2(13Z,16Z)), which were found to be elevated in NAC-responsive patients.

Alterations in individual metabolites reflect adaptation in metabolic pathways. We found that glutathione metabolism and glycine, serine and threonine metabolism were significantly enriched. Alterations in glycine, serine and threonine metabolic pathways have been reported in NAC response of breast cancer [19]. Glutathione (GSH) is an antioxidant metabolite capable of scavenging ROS [32]. Dysregulation of glutathione metabolism is present in bladder cancer patients [39]. Our previous study found that GSH reduction caused by overexpression of mir-218 increased the sensitivity of bladder cancer to cisplatin [40]. The enrichment of the glutathione metabolic pathway suggests that GSH level and oxidative stress status in NAC-sensitive patients may be different compared to NAC-resistant patients.

Enrichment analysis of metabolite-related genes demonstrated that DNA damage repair has crucial value. Defects in DNA repair genes could be used as biomarkers for predicting response to cisplatin-based NAC in bladder cancer and improve patients’ long-term survival after NAC [41,42]. Breast cancer patients with DNA repair gene mutations are more sensitive to NAC [43]. BAX is related to the differential metabolite hypoxanthine. BAX is involved in apoptosis, and the expression of BAX is more frequent in NAC responders among cervical cancer patients [44].

Overall, our study reported the metabolic profile of NAC sensitivity in patients with MIBC through untargeted metabolomics analysis of blood samples collected before the first chemotherapy. Additionally, we provided a theoretical basis from metabolic phenotype to potential genes for cisplatin resistance. However, there are some limitations in this study. First, this study was performed with a small number of samples. Second, the metabolic alterations caused by dietary patterns were not taken into account. Nevertheless, as the first exploration in metabolites reflected from NAC response in bladder cancer patients, our study also has implications for future studies. Conducting a more targeted metabolite analysis with a larger sample size is necessary, in the hope of improving the status of cisplatin resistance in MIBC patients.

## 4. Materials and Methods

### 4.1. Study Design

**Figure 7:**
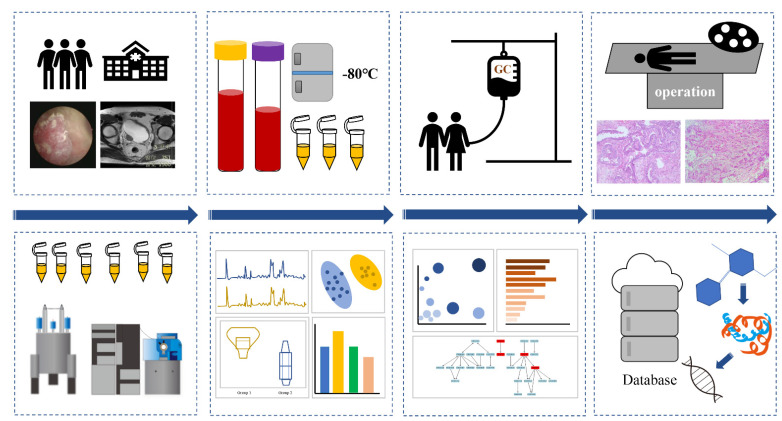
Workflows of this study, including the treatment of MIBC patients and the process of serum metabolomics.

Blood samples were prospectively collected before the first chemotherapy from patients who met NAC indications and agreed to receive NAC. ^1^H-NMR and UPLC-MS, two complementary detection methods, were used to develop serum metabolomic analysis at the Centre of Molecular Metabolism, Nanjing University of Science and Technology. All patients underwent magnetic resonance imaging (MRI) before the first chemotherapy to assess clinical stage. Tumour response to NAC were assessed by at least two pathologists. We considered patients with complete response (pCR: pT0N0) and partial response (pPR: pT1N0, pTaN0, or pTisN0) as NAC-sensitive, while patients with no response (≥pT2 or pN+) were considered NAC-resistant [8]. The workflow is shown in Figure 7.

### 4.2. Patients Population

Patients from January 2017 to December 2019 in the First Affiliated Hospital of Nanjing Medical University were recruited. Inclusion criteria were (1) clinical diagnosis as cT2-4aNxM0; (2) RC tolerated after clinical evaluation; (3) no history of chemotherapy or immunotherapy; (4) no history of metabolic diseases. Exclusion criteria were (1) incomplete clinical data; (2) not completed 2 cycles of NAC.

### 4.3. NAC Regimen

Patients were assigned to receive chemotherapy of gemcitabine (1.0 g/m^2^ on days 1 and 8) and cisplatin (70 mg/m^2^ evenly distributed over days 2 to 4) every 21 days for 2 cycles.

### 4.4. Serum Sample Collection

Fasting peripheral blood (3.5 mL) was collected in the morning before the first NAC treatment. The blood was centrifuged at 3000 rpm for 10 min at 4 °C. The serum was carefully absorbed into 1.5 mL centrifuge tubes and stored at −80 °C for metabolite detection.

### 4.5. Metabolomics Methods

#### 4.5.1. Chemicals and Reagents

Ammonium acetate, ammonia water, deuterium oxide (D2O, 99.9%) and sodium 3-trimethylsilyl propionic acid (TSP) were obtained from Sigma-Aldrich Co. (St Louis, MO, USA). Acetonitrile and methanol were purchased from Guangdong Guanghua SciTech Co. (Shantou, Guangdong, China). Solution preparation used ultrapure water (resistivity ≥18.25 MΩcm^−1^).

#### 4.5.2. Sample PreparationSample Preparation for ^1^H-NMR Spectroscopy

##### Sample Preparation for 1H-NMR Spectroscopy

A 600 μL amount of methanol was added into 300 μL serum and vortexed for 20 min. The supernatant was centrifuged, and the methanol was removed with a nitrogen blower. Then, samples were stored at −80 °C until the next day, followed by drying in a freeze-dryer. The lyophilized samples were dissolved in 600 μL D2O phosphate buffer (containing TSP as internal standard), then vortexed and centrifuged at 12,000 rpm for 5 min at 4 °C. Finally, the supernatant solution was transferred to 5 mm NMR tubes for ^1^H-NMR testing [45,46].

##### Sample Preparation for UPLC-MS

A mixture of acetonitrile and methanol at the ratio of 1:1 was prepared in advance. Into 100 μL serum, 400 μL mixture solution were added and vortexed for 30 s before freezing at −20 °C for 4 h. The samples were then centrifuged at 12,000 rpm for 12 min at 4 °C. The supernatants were absorbed and dried in a freeze dryer. Then, samples were dissolved with 100 μL precooling mixture solution (acetonitrile and water at the radio of 1:1), treated with ultrasound in ice water for 5 min, vortexed for 30 s, and centrifuged at 12,000 rpm for 5 min at 4 °C. Finally, the supernatant solution was transferred to tubes for UPLC-MS testing.

### 4.5.3. Data Pre-Processing

#### ^1^H-NMR Spectroscopy and Data Pre-Processing

The methods of ^1^H-NMR spectroscopy and data pre-processing were as described in previous articles [45,47]. Briefly, the ^1^H-NMR spectra were obtained by Bruker AVANCE Ⅲ 500 MHz spectrometer (Bruker GmbH, Karlsruhe, Germany). The NMR spectra of serum samples were collected at 298 K using modified transverse relaxation edited Call-Purcell-Meiboom-Gill (CPMG) sequence (90 (τ-180-τ) n-acquisition), with a total spin echo delay (2nτ) of 10 ms to suppress the signals of proteins. Then, free induction decay (FID) was multiplied with an exponential window function corresponding to the 0.5 Hz line spreading factor before performing Fourier transform. Phase and baseline of ^1^H-NMR data were adjusted by Bruker TopSpin Software (version 3.5, Bruker). Mestre C (version 4.9.9.6, Mestrelab Research SL) was used to export the adjusted ^1^H-NMR spectra into ASCII files, which were then imported into “R” software for multivariate data analysis.

#### UPLC-MS Condition and Data Pre-Processing

A UPLC BEH Amide column (1.7 μm, 2.1 × 100 mm, Waters) was used for UPLC-MS (Triple TOF 5600+ MS, AB SCIEX, USA). Mobile phase A was 20 mM ammonia plus 25 mM ammonium acetate aqueous solution (500 mL pure water with 0.963 g ammonium acetate and 0.774 mL ammonia), and mobile phase B was acetonitrile. The gradient procedure is shown in Table S1. The flow rate was 0.3 mL/min, and the column temperature was set as 40 °C. One QC sample was put into every three samples.

The MS was performed in negative ion mode with an electrospray (ESI) ion source. The bombardment energy was set at 35 ± 15 eV, and the cumulative time of each product ion was 50 ms. Firstly, ProteoWizard software (https://proteowizard.sourceforge.io/ (accessed on 31 December 2021)) was used to convert the original MS data into a common mzXML format. Then, a data matrix consisting of retention time (RT), mass/charge ratio (M/z) and peak intensity was obtained by pre-processing the data. Compound peaks were annotated by MS database and “R” software ((http://cran.r-project.org/ (accessed on 31 December 2021)) package XCMS. Features with CV over 15% in pooled QC samples were removed, and the missing values were input by random forest imputation. Finally, PCA and OPLS-DA were performed on the data obtained from sample names, corresponding compound of each peak, and peak area of each compound after normalization.

#### 4.5.4. Multivariate Statistical Analysis

PCA and OPLS-DA are commonly used in multivariate statistics of metabolomics. PCA, an unsupervised exploratory analysis, uses dimensionality reduction to identify the overall distribution of the samples. OPLS-DA is a supervised recognition method that can better clusters between the two groups by filtering through irrelevant effects [48]. Fold change was calculated by integrating the area ratio of metabolites, and the associated *p*-values were calculated and corrected by the Benjamin–Hochberg method. All statistical analyses were run in the “R” software.

#### 4.5.5. Metabolic Pathway and Enrichment Analysis

Metabolic pathway analysis and metabolite set enrichment analysis (MSEA) was performed in Metaboanalyst5.0 (https://www.metaboanalyst.ca/ (accessed on 16 February 2022)). Enrichment analysis and hallmark analysis were run in the “R” software.

## 5. Conclusions

Serum metabolic profiles of NAC sensitivity are significantly different in bladder cancer patients. Glycine, hypoxanthine, taurine and glutamine may be potential biomarkers for contributing to clinical treatment. Targeting amino acid metabolic pathways is expected to be a new direction to improve the sensitivity of NAC in bladder cancer.

## Figures and Tables

**Table 1:** Patient baseline characteristics.

	NAC-Sensitive	NAC-Resistant
Patient number	6	12
Sex, n (%)		
Male	6 (100%)	12 (100%)
Female	0 (0)	0 (0)
Age, median (range)	66.5 (39–75)	64.5 (49–77)
BMI, M ± SD (kg/m^2^)	24.9 ± 4.2	23.5 ± 2.4
Clinical T stage, n (%)		
T2	4	5
T3	2	6
T4	0	1
Pathological T stage		
T0	2	0
T1	4	0
T2	0	6
T3	0	5
T4	0	1
Smoking, n (%)		
Yes	2 (33.3%)	8 (66.7%)
No	4 (66.7%)	4 (33.3%)
Chemical exposure, n (%)		
Yes	0 (0)	1 (8.3%)
No	6 (100%)	11 (91.7%)

**Table 2:** Identified metabolites between the two groups by ^1^H-NMR.

Metabolites	Sensitive vs. Resistant	
	Log2(FC)	*p*
2-Hydroxybutyrate	−0.11	
Isoleucine	−0.08	
2-Hydroxy-3-methylvalerate	−0.32	**
Leucine	−0.32	
Valine	−0.22	
3-Methyl-2-oxovalerate	−0.44	**
3-Hydroxybutyrate	−0.54	*
Lactate	−0.15	
Alanine	−0.8	**
Lysine	−0.06	
Acetate	−0.08	
Glutamate	−0.66	*
Pyruvate	−2.06	**
Pyroglutamate	−1.07	***
Glutamine	0.69	*
Ornithine	0.01	
Choline	−0.31	
Carnitine	−0.01	
Betaine	0.89	
Trimethylamine-N-oxide	0.04	
Taurine	1.23	*
Glycerol	0.05	
Glycine	−0.82	**
Creatine	−0.73	
Tyrosine	0.03	
Histidine	0.07	
Phenylalanine	−0.06	
Hypoxanthine	−0.48	*
Formate	0.3	

*: *p* < 0.05, **: *p* < 0.01, ***: *p* < 0.001.

**Table 3:** Metabolic pathway analysis of metabolites identified by ^1^H-NMR.

Pathway Name	Matched Metabolites	Raw p (× 10 ^−^³)	−log10(p)	FDR (× 10 ^−^³)	Impact
Alanine, aspartate and glutamate metabolism	4/28	0.02	4.7786	1.21	0.3109
Glyoxylate and dicarboxylate metabolism	4/32	0.03	4.5394	1.21	0.10582
D-glutamine and D-glutamate metabolism	2/6	0.55	3.256	8.16	0.5
Glutathione metabolism	3/28	0.58	3.2345	8.16	0.11548
Arginine biosynthesis	2/14	3.27	2.4851	39.27	0.11675
Glycine, serine and threonine metabolism	2/33	17.78	1.75	149.37	0.24577
Taurine and hypotaurine metabolism	1/8	50.57	1.2961	283.21	0.42857

The table contains a partial results of pathway analysis. The impact is the pathway impact value calculated from pathway topology analysis.

**Table 4:** Metabolic pathway analysis of metabolites identified by UPLC-MS.

Pathway Name	Matched Metabolites	Raw p	−log10(p)	FDR	Impact
Glutathione metabolism	4/28	0.02	1.6362	1	0.12042
Glyoxylate and dicarboxylate metabolism	4/32	0.04	1.4433	1	0.4127
Glycine, serine and threonine metabolism	4/33	0.04	1.4001	1	0.24577
Taurine and hypotaurine metabolism	1/8	0.28	0.5542	1	0.42857

The table contains partial results of pathway analysis. The impact is the pathway impact value calculated from pathway topology analysis.

## Data Availability

All data generated or analysed during this study are included in this published article and its supplementary information files. Further enquiries can be directed to the corresponding authors.

## Supplementary Materials

The following supporting information can be downloaded at: https://www.mdpi.com/article/10.3390/metabo12060558/s1. Figure S1 PCA score plot of ^1^H-NMR. Figure S2 PCA (A) and OPLS-DA (B) analysis of the data obtained from UPLC-MS. Figure S3 Other significant metabolites identified by UPLC-MS between the two groups. Figure S4 The genes associated with these pathways and metabolites. Table S1 Gradient elution table of UPLC-MS. Table S2 Metabolites corresponding to each violin diagram.
