# Supplementary material for: Metabolic Profiling of Bladder Cancer Patients’ Serum Reveals Their Sensitivity to Neoadjuvant Chemotherapy
Source: Metabolites. 2022 Jun 17;12(6):558. doi: 10.3390/metabo12060558 (PMC9229374; doi:10.3390/metabo12060558)

Figure S1 PCA score plot of ^{1}H-NMR. Each point is each sample. Different groups are in different colors, with circles representing the 95% confidence interval.

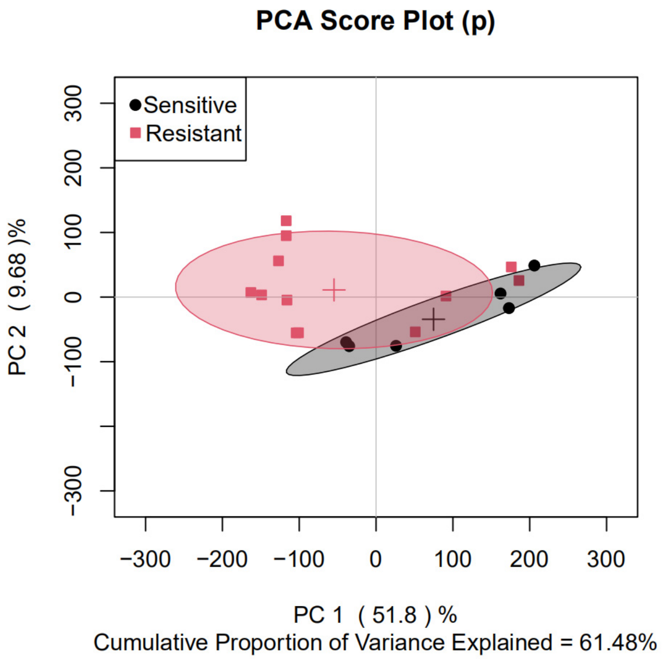

Figure S2 PCA(A) and OPLS-DA(B) analysis of the data achieved from UPLC-MS. Each point is each sample. Different groups are in different colors, with circles representing the 95% confidence interval.

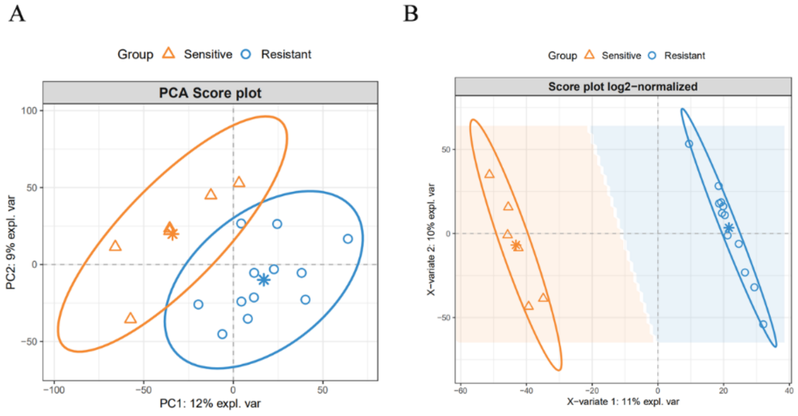

Figure S3 Other significant metabolites identified by UPLC-MS between the two groups.

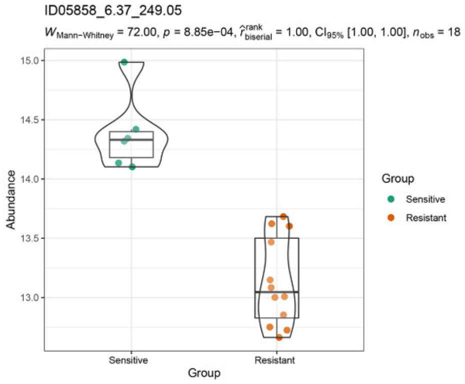
ID05858_6.37_249.05
$W_{\text{Mann-Whitney}}$ = 72.00, p = 8.85e−04, $\hat{r}_{\text{biserial}}^{\text{rank}}$ = 1.00, $\text{CI}_{95\%}$ [1.00, 1.00], n_{obs} = 18
Abundance
15.0
14.5
14.0
13.5
13.0
Sensitive
Resistant
Group
Group
Sensitive
Resistant

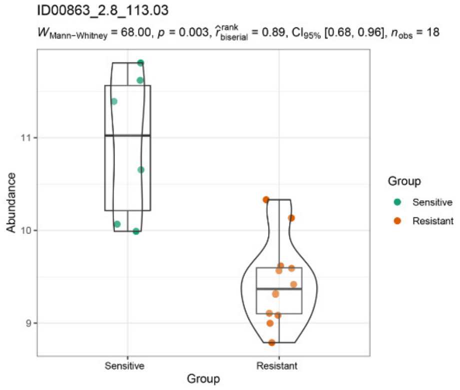
ID00863_2.8_113.03
$W_{\text{Mann-Whitney}}$ = 68.00, p = 0.003, $\hat{r}_{\text{biserial}}^{\text{rank}}$ = 0.89, $\text{CI}_{95\%}$ [0.68, 0.96], n_{obs} = 18
Abundance
11
10
9
Sensitive
Resistant
Group
Group
Sensitive
Resistant

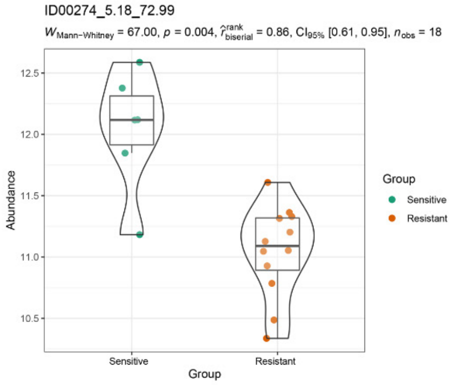
ID00274_5.18_72.99
$W_{\text{Mann-Whitney}}$ = 67.00, p = 0.004, $\hat{r}_{\text{biserial}}^{\text{rank}}$ = 0.86, $\text{CI}_{95\%}$ [0.61, 0.95], n_{obs} = 18
Abundance
12.5
12.0
11.5
11.0
10.5
Sensitive
Resistant
Group
Group
Sensitive
Resistant

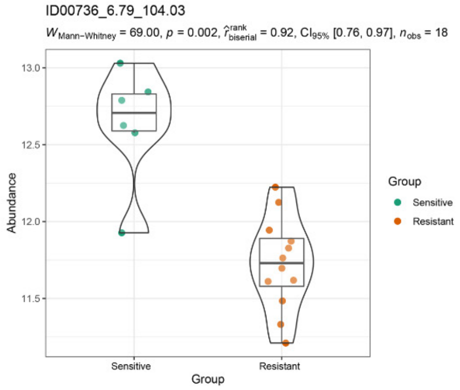
ID00736_6.79_104.03
$W_{\text{Mann-Whitney}}$ = 69.00, p = 0.002, $\hat{r}_{\text{biserial}}^{\text{rank}}$ = 0.92, $\text{CI}_{95\%}$ [0.76, 0.97], n_{obs} = 18
Abundance
13.0
12.5
12.0
11.5
Sensitive
Resistant
Group
Group
Sensitive
Resistant

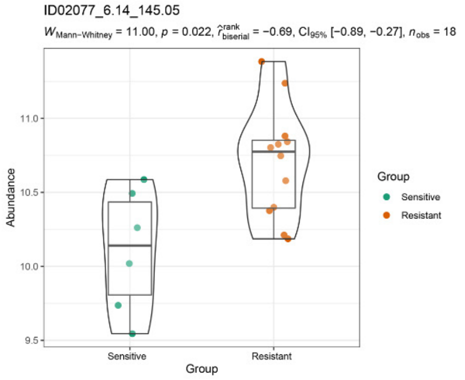
ID02077_6.14_145.05
$W_{\text{Mann-Whitney}}$ = 11.00, p = 0.022, $\hat{r}_{\text{biserial}}^{\text{rank}}$ = −0.69, $\text{CI}_{95\%}$ [−0.89, −0.27], n_{obs} = 18
Abundance
11.0
10.5
10.0
9.5
Sensitive
Resistant
Group
Group
Sensitive
Resistant

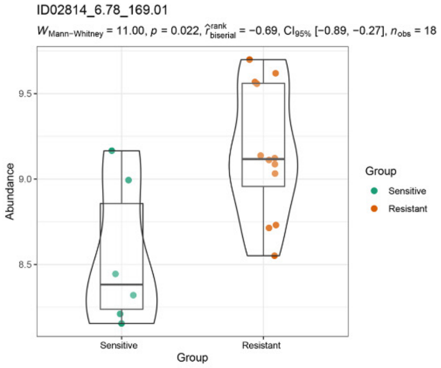
ID02814_6.78_169.01
$W_{\text{Mann-Whitney}}$ = 11.00, p = 0.022, $\hat{r}_{\text{biserial}}^{\text{rank}}$ = −0.69, $\text{CI}_{95\%}$ [−0.89, −0.27], n_{obs} = 18
Abundance
9.5
9.0
8.5
Sensitive
Resistant
Group
Group
Sensitive
Resistant

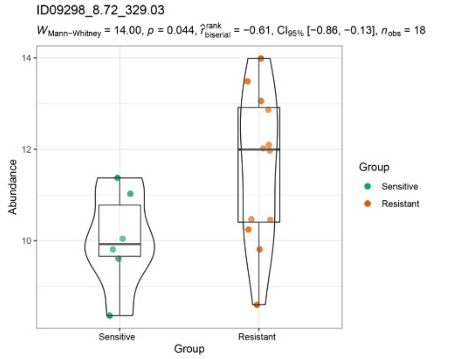
ID09298_8.72_329.03
$W_{\text{Mann-Whitney}}$ = 14.00, p = 0.044, $\hat{r}_{\text{biserial}}^{\text{rank}}$ = −0.61, $\text{CI}_{95\%}$ [−0.86, −0.13], n_{obs} = 18
Abundance
14
12
10
Sensitive
Resistant
Group
Group
Sensitive
Resistant

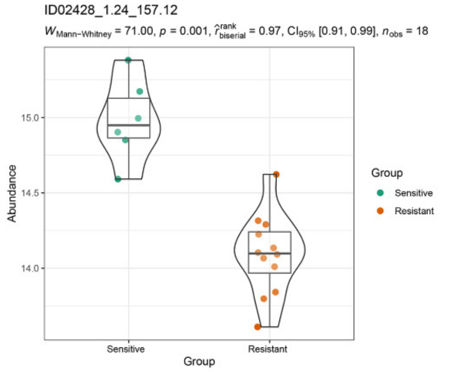
ID02428_1.24_157.12
$W_{\text{Mann-Whitney}}$ = 71.00, p = 0.001, $\hat{r}_{\text{biserial}}^{\text{rank}}$ = 0.97, $\text{CI}_{95\%}$ [0.91, 0.99], n_{obs} = 18
Abundance
15.0
14.5
14.0
Sensitive
Resistant
Group
Group
Sensitive
Resistant

Figure S4 The genes that associated with these pathways and metabolites. Red represents genes and green represents pathways.

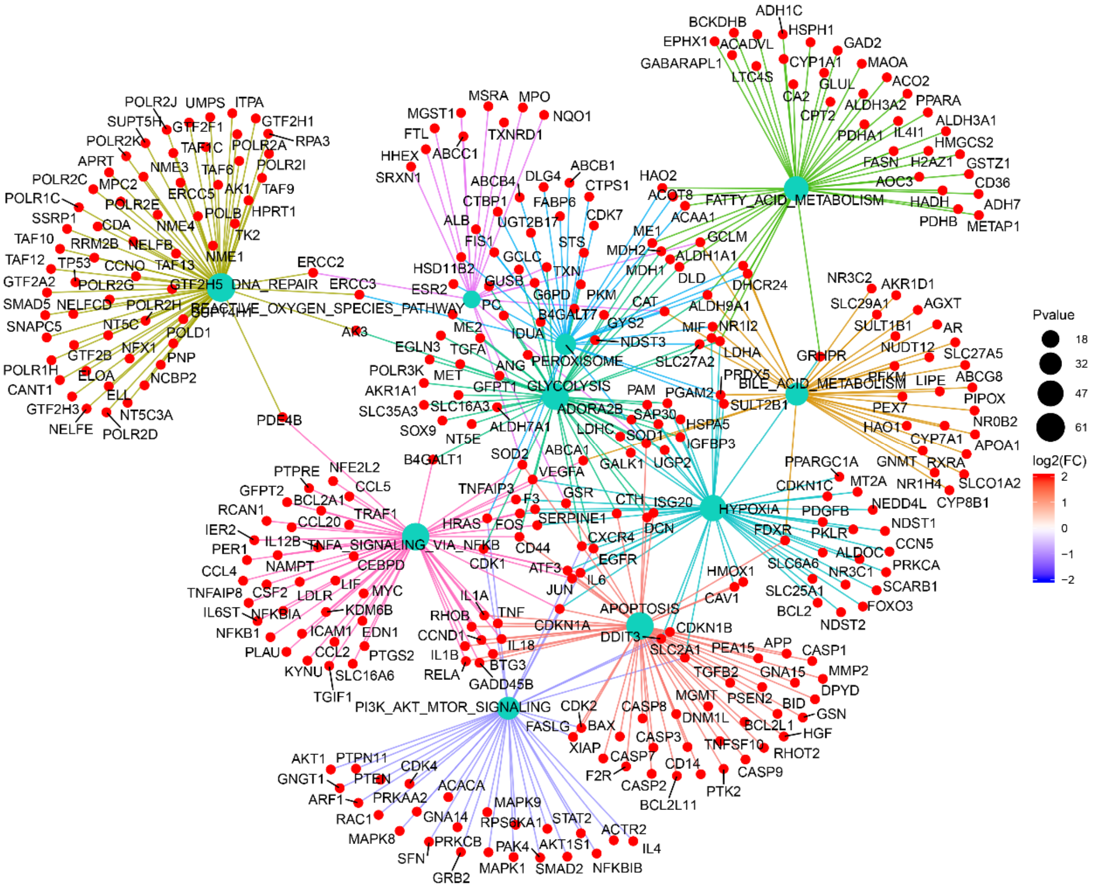

Supplement: Supplementary file 1 [file metabolites-12-00558-s001.zip › Supplement Figures.pdf]
