# Supplementary material for: Metabolic Profiling of Bladder Cancer Patients’ Serum Reveals Their Sensitivity to Neoadjuvant Chemotherapy
Source: Metabolites. 2022 Jun 17;12(6):558. doi: 10.3390/metabo12060558 (PMC9229374; doi:10.3390/metabo12060558)
Supplement: Supplementary file 1 [file metabolites-12-00558-s001.zip › Supplement Tables.pdf]

Table S1 Gradient elution table of UPLC-MS

t/min	A/%	B/%
0	5	95
6	30	70
7	35	65
10	60	40
10.1	5	95
15	5	95

Table S2 Metabolites corresponding to each violin diagram.

ID02315_9.91_154.06	L-Histidine
ID01385_6.9_131.08	Ornithine
ID06320_6.7_263.01	L-Cystine
ID04424_3.66_215.02	Citric acid
ID05858_6.37_249.05	gamma-Glutamylcysteine
ID00863_2.8_113.03	Uracil
ID00274_5.18_72.99	Glyoxylic acid
ID00736_6.79_104.03	Serine
ID02077_6.14_145.05	Hexanedioic acid
ID02814_6.78_169.01	Gallic acid
ID09298_8.72_329.03	2,3-Dihydroxybenzoic acid
ID02428_1.24_157.12	Allantoin
